# Redetermination of hepta­potassium nona­hydrogen bis­[α-hexa­molybdoplatinate(IV)] undeca­hydrate

**DOI:** 10.1107/S1600536810008639

**Published:** 2010-03-13

**Authors:** Uk Lee, Hea-Chung Joo, Ki-Min Park

**Affiliations:** a Department of Chemistry, Pukyong National University, 599-1 Daeyeon 3-dong, Nam-gu, Busan 608-737, Republic of Korea; b Department of Chemistry, Dongeui University, San 24 Kaya-dong Busanjin-gu, Busan 614-714, Republic of Korea; c The Research Institute of Natural Science, Gyeongsan National University, Jinju 660-701, Republic of Korea

## Abstract

Previously reported at a temperature of 298 (2) K [Lee & Joo (2007 ▶). *Acta Cryst*. E**63**, i11–i13], the title compound, K_7_[H_9_α-Pt_2_Mo_12_O_48_]·11H_2_O or K_7_[H_4.5_α-PtMo_6_O_24_]_2_·11H_2_O, was redetermined at 146 (2) K in order to determine whether the H atom in the hydrogen bond that crosses the center of symmetry was located at the center of symmetry or disordered around it as assumed in the previous study. During the present low-temperature study it was found on the center of symmetry. One water molecule shows half-occupancy.

## Related literature

For the crystal structure of K_3.5_[H_4.5_α-PtMo_6_O_24_]·5.5H_2_O, see: Lee & Joo (2007 ▶). For related structures, see: Lee & Sasaki (1994 ▶); Joo *et al.* (1994 ▶); Lee & Joo (2006*a* ▶,*b* ▶).
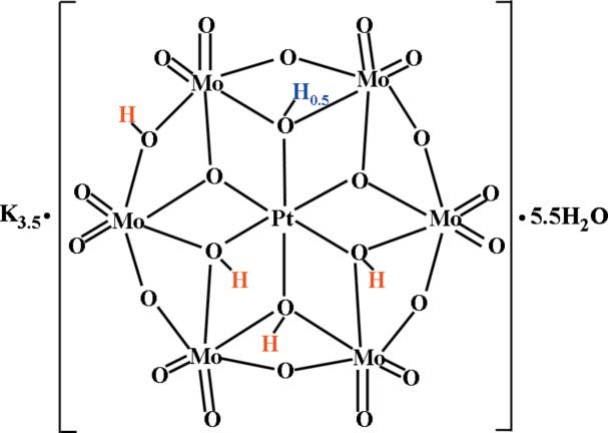


## Experimental

### 

#### Crystal data

K_7_[H_4.5_PtMo_6_O_24_]_2_·11H_2_O
*M* *_r_* = 2790.39
Triclinic, 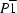

*a* = 10.0430 (3) Å
*b* = 12.1512 (4) Å
*c* = 12.4498 (4) Å
α = 67.792 (1)°
β = 68.542 (1)°
γ = 83.465 (2)°
*V* = 1308.58 (7) Å^3^
*Z* = 1
Mo *K*α radiation
μ = 8.78 mm^−1^
*T* = 148 K
0.15 × 0.09 × 0.06 mm

#### Data collection

Bruker SMART APEXII CCD diffractometer
Absorption correction: multi-scan (*SADABS*; Bruker, 2009 ▶) *T*_min_ = 0.353, *T*_max_ = 0.621
22108 measured reflections
5699 independent reflections
5599 reflections with *I* > 2σ(*I*)
*R*_int_ = 0.031

#### Refinement

*R*[*F* ^2^ > 2σ(*F* ^2^)] = 0.021
*wR*(*F* ^2^) = 0.056
*S* = 1.23
5699 reflections
430 parameters
20 restraints
H atoms treated by a mixture of independent and constrained refinement
Δρ_max_ = 0.88 e Å^−3^
Δρ_min_ = −2.50 e Å^−3^

### 

Data collection: *APEX2* (Bruker, 2009 ▶); cell refinement: *SAINT* (Bruker, 2009 ▶); data reduction: *SAINT*; program(s) used to solve structure: *SHELXS97* (Sheldrick, 2008 ▶); program(s) used to refine structure: *SHELXL97* (Sheldrick, 2008 ▶); molecular graphics: *ORTEP-3* (Farrugia, 1997 ▶) and *DIAMOND* (Brandenburg, 1998 ▶); software used to prepare material for publication: *SHELXL97*.

## Supplementary Material

Crystal structure: contains datablocks global, I. DOI: 10.1107/S1600536810008639/br2139sup1.cif

Structure factors: contains datablocks I. DOI: 10.1107/S1600536810008639/br2139Isup2.hkl

Additional supplementary materials:  crystallographic information; 3D view; checkCIF report

## Figures and Tables

**Table 1:** Hydrogen-bond geometry (Å, °)

*D*—H⋯*A*	*D*—H	H⋯*A*	*D*⋯*A*	*D*—H⋯*A*
O2*C*—H2⋯O24*T*^i^	0.87 (3)	1.70 (3)	2.561 (3)	168 (5)
O3*C*—H3⋯O5*W*^ii^	0.89 (3)	1.67 (3)	2.552 (4)	177 (5)
O4*C*—H4⋯O13*T*^i^	0.89 (5)	1.67 (5)	2.562 (3)	176 (5)
O6*C*—H6⋯O6*C*^i^	1.28	1.28	2.553 (3)	180
O11*B*—H11⋯O7*B*^i^	0.90 (5)	1.93 (5)	2.826 (3)	172 (4)

Symmetry codes: (i) 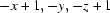; (ii) 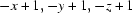.

**Fig. 1.**
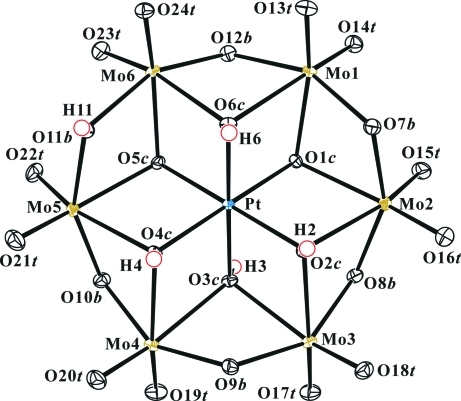
The polyanion structure in the title compound. Displacement ellipsoids are drawn at the 50 % probability level for non-H atoms. H atoms are presented as a small spheres of arbitrary radius.

**Fig. 2.**
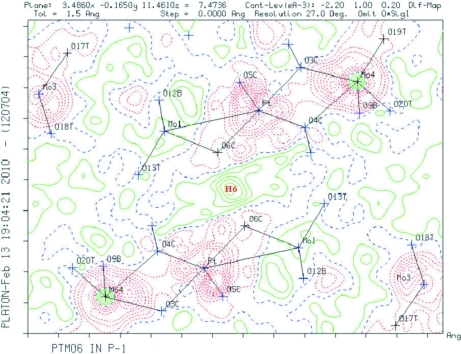
Difference Fourier map around H6 atom where atom H6 is absent.

**Fig. 3.**
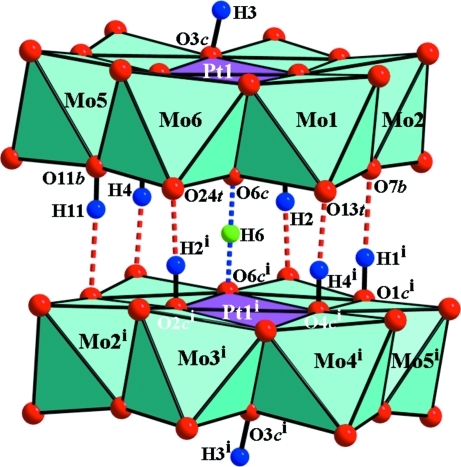
Polyhedral view of the heteropolyanion in the title compound with O–H···O contacts of the inter-anion hydrogen bonds shown as red dashed lines. [Symmetry code: (i) - x + 1, - y, - z + 1.]

## supplementary crystallographic information

### Comment

The α-β-α geometrical isomerization according to the stepwise protonation in the [PtMo_6_O_24_]^8-^ polyoxometalate species is very unusual phenomenon, viz., ([H_3.5_α-PtMo_6_O_24_]^4.5-^ (Lee & Sasaki, 1994), [H_4_β-PtMo_6_O_24_]^4-^ (Lee & Sasaki, 1994; Joo *et al.*, 1994) and [H_4.5_α-PtMo_6_O_24_]^3.5-^ (Lee & Sasaki, 1994; Lee & Joo, 2007).

This study was carried out to identify the position of the hydrogen atom that lies close to the center of symmetry. The structure of the title compound has been discussed in detail (Lee & Joo, 2007). Fig. 1 shows the structure of the polyanion. The O atoms of the clusters were designated as O*t*(terminal Mo═O atom), O*b* (O bridged µ_2_-O atom), and O*c* (µ_3_-O atom). The protonated O atoms in the polyanion were identified by the location in difference Fourier maps of the H atoms bound to O atoms and local structural features as seen previously (Lee & Joo, 2006a,b and Table 1). Fig. 2 shows a symmetrical electron density map around H6 atom. The position of H6 is (1/2, 0, 1/2). The distance of O6c–H6 and O6c···.O6c^i^ are 1.28Å and 2.553 (5) Å, and the bond angle of O6*c*–H6–O6*c*^i^ is 180 ° (Table 1 & Fig. 3).

The position of H6 was not found on the diffenence map in the previoius report (Lee & Joo, 2007). Therefore the H atom was considered as having positional disorder. The K2 ion is located on the inversion center. As a result the number of K^+^ ion is 7 and H^+^ ion is 9 in the unit cell.

The K^+^ ions are variously coordinated by O atoms as [K1(O_b_)(O_t_)_2_(O_w_)_4_]^+^, [K2(O_b_)_2_(O_t_)_6_]^+^, [K3(O_b_)(O_t_)_5_(O_w_)_2_]^+^, and [K4(O_t_)_5_(O_w_)_2_]^+^, respectively.

### Experimental

Crystals of title compound were prepared by the reaction of K_2_MoO_4_.2H_2_O and K_2_Pt(OH)_6_ at pH 2.85 as described in a previous report (Lee & Sasaki, 1994).

### Refinement

All H atoms in the polyanion were positioned in a difference Fourier maps and refined freely except H2 and H3. H2 and H3 refined with a distances restraint of O–H = 0.85 (3) Å. The H atoms of all O_w_ water molecules were placed in calculated positions with a distances restraint of O–H = 0.85 (3) Å. Their displacement parameters were freely refined except the O6_w_ water molecule. The reasonable termal ellipsoid of O6_w_ was obtained by half occupancy. The H atoms of O6_w_ were included in the refinement with *U*_iso_(H) = 1.5 *U*_eq_(O).

### Crystal data

**Table:**

K_7_[H_4.5_PtMo_6_O_24_]_2_·11H_2_O	*Z* = 1
*M**_r_* = 2790.39	*F*(000) = 1296.0
Triclinic, *P*1	*D*_x_ = 3.541 Mg m^−^^3^
Hall symbol: -P 1	Mo *K*α radiation, λ = 0.71073 Å
*a* = 10.0430 (3) Å	Cell parameters from 9000 reflections
*b* = 12.1512 (4) Å	θ = 2.5–28.3°
*c* = 12.4498 (4) Å	µ = 8.78 mm^−^^1^
α = 67.792 (1)°	*T* = 148 K
β = 68.542 (1)°	Block, pale yellow
γ = 83.465 (2)°	0.15 × 0.09 × 0.06 mm
*V* = 1308.58 (7) Å^3^	

### Data collection

**Table:**

Bruker SMART APEXII CCD diffractometer	5699 independent reflections
Radiation source: Rotating Anode	5599 reflections with *I* > 2σ(*I*)
Bruker HELIOS graded multilayer optics	*R*_int_ = 0.031
Detector resolution: 10.0 pixels mm^-1^	θ_max_ = 27.0°, θ_min_ = 1.8°
φ and ω scans	*h* = −12→12
Absorption correction: multi-scan (*SADABS*; Bruker, 2009)	*k* = −15→15
*T*_min_ = 0.353, *T*_max_ = 0.621	*l* = −15→15
22108 measured reflections	

### Refinement

**Table:**

Refinement on *F*^2^	Primary atom site location: structure-invariant direct methods
Least-squares matrix: full	Secondary atom site location: difference Fourier map
*R*[*F*^2^ > 2σ(*F*^2^)] = 0.021	Hydrogen site location: difference Fourier map
*wR*(*F*^2^) = 0.056	H atoms treated by a mixture of independent and constrained refinement
*S* = 1.23	*w* = 1/[σ^2^(*F*_o_^2^) + (0.0236*P*)^2^ + 1.1372*P*] where *P* = (*F*_o_^2^ + 2*F*_c_^2^)/3
5699 reflections	(Δ/σ)_max_ = 0.001
430 parameters	Δρ_max_ = 0.88 e Å^−^^3^
20 restraints	Δρ_min_ = −2.50 e Å^−^^3^

### Special details

**Table:**

Geometry. All esds (except the esd in the dihedral angle between two l.s. planes) are estimated using the full covariance matrix. The cell esds are taken into account individually in the estimation of esds in distances, angles and torsion angles; correlations between esds in cell parameters are only used when they are defined by crystal symmetry. An approximate (isotropic) treatment of cell esds is used for estimating esds involving l.s. planes.
Refinement. Refinement of *F*^2^ against ALL reflections. The weighted *R*-factor wR and goodness of fit *S* are based on *F*^2^, conventional *R*-factors *R* are based on *F*, with *F* set to zero for negative *F*^2^. The threshold expression of *F*^2^ > σ(*F*^2^) is used only for calculating *R*-factors(gt) etc. and is not relevant to the choice of reflections for refinement. *R*-factors based on *F*^2^ are statistically about twice as large as those based on *F*, and *R*- factors based on ALL data will be even larger.

### Fractional atomic coordinates and isotropic or equivalent isotropic displacement parameters (Å^2^)

**Table:**

	*x*	*y*	*z*	*U*_iso_*/*U*_eq_	Occ. (<1)
Pt	0.370508 (12)	0.214544 (10)	0.496464 (11)	0.00637 (5)	
Mo1	0.70622 (3)	0.22945 (2)	0.32045 (3)	0.00847 (7)	
Mo2	0.47058 (3)	0.32387 (3)	0.19019 (3)	0.00951 (7)	
Mo3	0.12269 (3)	0.31421 (2)	0.35851 (3)	0.00933 (7)	
Mo4	0.01778 (3)	0.22408 (2)	0.66624 (3)	0.00926 (7)	
Mo5	0.26446 (3)	0.14143 (3)	0.79903 (3)	0.00971 (7)	
Mo6	0.61144 (3)	0.14446 (2)	0.62239 (3)	0.00808 (7)	
K1	0.07055 (10)	0.42124 (8)	0.85759 (9)	0.0273 (2)	
K2	0.0000	0.0000	0.5000	0.0355 (4)	
K3	0.59498 (11)	0.36460 (8)	0.82891 (9)	0.0313 (2)	
K4	0.26045 (10)	0.09021 (8)	0.12674 (8)	0.0281 (2)	
O1C	0.5151 (2)	0.3258 (2)	0.3496 (2)	0.0090 (5)	
O2C	0.3163 (3)	0.1907 (2)	0.3667 (2)	0.0097 (5)	
H2	0.316 (5)	0.118 (3)	0.370 (4)	0.039 (14)*	
O3C	0.2066 (3)	0.3296 (2)	0.4991 (2)	0.0100 (5)	
H3	0.239 (5)	0.401 (3)	0.484 (5)	0.051 (16)*	
O4C	0.2191 (2)	0.1077 (2)	0.6475 (2)	0.0088 (5)	
H4	0.206 (5)	0.034 (4)	0.652 (4)	0.036 (13)*	
O5C	0.4239 (2)	0.2395 (2)	0.6249 (2)	0.0095 (5)	
O6C	0.5309 (3)	0.1025 (2)	0.4921 (2)	0.0098 (5)	
H6	0.5000	0.0000	0.5000	0.07 (3)*	
O7B	0.6133 (3)	0.1997 (2)	0.2207 (2)	0.0122 (5)	
O8B	0.2979 (2)	0.4051 (2)	0.2462 (2)	0.0114 (5)	
O9B	0.0238 (2)	0.1989 (2)	0.5211 (2)	0.0119 (5)	
O10B	0.1262 (3)	0.2514 (2)	0.7562 (2)	0.0127 (5)	
O11B	0.4359 (3)	0.0325 (2)	0.7600 (2)	0.0124 (5)	
H11	0.429 (5)	−0.044 (4)	0.767 (5)	0.042 (14)*	
O12B	0.7099 (2)	0.2530 (2)	0.4686 (2)	0.0116 (5)	
O13T	0.8149 (3)	0.1062 (2)	0.3315 (2)	0.0139 (5)	
O14T	0.8155 (3)	0.3469 (2)	0.2130 (2)	0.0145 (5)	
O15T	0.5792 (3)	0.4457 (2)	0.0911 (2)	0.0168 (5)	
O16T	0.4268 (3)	0.2674 (2)	0.0998 (2)	0.0168 (6)	
O17T	0.0066 (3)	0.4276 (2)	0.3516 (3)	0.0170 (6)	
O18T	0.0983 (3)	0.2410 (2)	0.2724 (2)	0.0144 (5)	
O19T	−0.0931 (3)	0.3406 (2)	0.6643 (3)	0.0178 (6)	
O20T	−0.0825 (3)	0.1011 (2)	0.7766 (2)	0.0171 (6)	
O21T	0.1625 (3)	0.0179 (2)	0.9083 (2)	0.0194 (6)	
O22T	0.3249 (3)	0.2023 (2)	0.8760 (2)	0.0160 (5)	
O23T	0.6388 (3)	0.1967 (2)	0.7219 (2)	0.0146 (5)	
O24T	0.7139 (2)	0.0163 (2)	0.6320 (2)	0.0122 (5)	
O1W	0.0212 (4)	0.1809 (3)	1.0680 (3)	0.0361 (8)	
H1A	−0.039 (6)	0.190 (5)	1.132 (4)	0.08 (2)*	
H1B	−0.010 (6)	0.114 (4)	1.070 (6)	0.08 (2)*	
O2W	0.7580 (6)	0.2330 (4)	0.9609 (4)	0.0773 (17)	
H2A	0.738 (8)	0.214 (7)	1.039 (3)	0.13 (3)*	
H2B	0.812 (5)	0.181 (4)	0.942 (5)	0.065 (19)*	
O3W	0.3643 (3)	0.4685 (3)	0.6662 (3)	0.0297 (7)	
H3A	0.421 (4)	0.528 (3)	0.642 (5)	0.046 (15)*	
H3B	0.415 (5)	0.413 (3)	0.652 (5)	0.052 (17)*	
O4W	0.1450 (3)	0.4420 (3)	1.0368 (3)	0.0232 (6)	
H4A	0.226 (3)	0.470 (4)	1.000 (4)	0.043 (15)*	
H4B	0.145 (5)	0.382 (3)	1.100 (3)	0.037 (14)*	
O5W	0.7096 (3)	0.4622 (2)	0.5447 (3)	0.0199 (6)	
H5A	0.782 (4)	0.418 (4)	0.548 (4)	0.046 (15)*	
H5B	0.707 (4)	0.478 (4)	0.474 (3)	0.023 (13)*	
O6W	0.4612 (8)	−0.0364 (7)	0.0153 (7)	0.044 (2)	0.50
H6A	0.534 (8)	−0.080 (9)	0.005 (11)	0.066*	0.50
H6B	0.410 (11)	−0.064 (11)	−0.010 (11)	0.066*	0.50

### Atomic displacement parameters (Å^2^)

**Table:**

	*U*^11^	*U*^22^	*U*^33^	*U*^12^	*U*^13^	*U*^23^
Pt	0.00609 (7)	0.00591 (7)	0.00734 (8)	0.00024 (5)	−0.00257 (6)	−0.00247 (6)
Mo1	0.00692 (14)	0.00822 (13)	0.01040 (15)	0.00071 (10)	−0.00275 (11)	−0.00396 (12)
Mo2	0.00948 (14)	0.01008 (14)	0.00836 (15)	−0.00072 (10)	−0.00320 (12)	−0.00235 (12)
Mo3	0.00878 (14)	0.00899 (14)	0.01138 (16)	0.00063 (10)	−0.00517 (12)	−0.00346 (12)
Mo4	0.00725 (14)	0.00911 (14)	0.01092 (15)	0.00039 (10)	−0.00240 (12)	−0.00391 (12)
Mo5	0.00970 (14)	0.01067 (14)	0.00813 (15)	0.00112 (11)	−0.00255 (12)	−0.00355 (12)
Mo6	0.00793 (14)	0.00677 (13)	0.01079 (15)	0.00094 (10)	−0.00482 (12)	−0.00325 (12)
K1	0.0422 (5)	0.0217 (4)	0.0297 (5)	0.0106 (4)	−0.0209 (4)	−0.0167 (4)
K2	0.0150 (6)	0.0269 (7)	0.0648 (10)	−0.0064 (5)	0.0054 (6)	−0.0334 (7)
K3	0.0470 (6)	0.0201 (4)	0.0281 (5)	0.0015 (4)	−0.0120 (5)	−0.0114 (4)
K4	0.0356 (5)	0.0302 (5)	0.0164 (4)	−0.0166 (4)	−0.0084 (4)	−0.0022 (4)
O1C	0.0081 (11)	0.0091 (11)	0.0080 (12)	−0.0020 (9)	−0.0016 (9)	−0.0017 (10)
O2C	0.0123 (12)	0.0082 (11)	0.0106 (13)	0.0000 (9)	−0.0056 (10)	−0.0041 (10)
O3C	0.0088 (12)	0.0089 (11)	0.0130 (13)	−0.0002 (9)	−0.0051 (10)	−0.0033 (10)
O4C	0.0078 (11)	0.0066 (11)	0.0089 (12)	−0.0021 (9)	−0.0010 (10)	−0.0007 (10)
O5C	0.0097 (11)	0.0120 (11)	0.0097 (12)	0.0009 (9)	−0.0050 (10)	−0.0057 (10)
O6C	0.0080 (11)	0.0093 (11)	0.0123 (13)	0.0008 (9)	−0.0037 (10)	−0.0042 (10)
O7B	0.0110 (12)	0.0144 (12)	0.0137 (13)	0.0009 (9)	−0.0044 (10)	−0.0077 (11)
O8B	0.0111 (12)	0.0107 (11)	0.0127 (13)	0.0000 (9)	−0.0058 (10)	−0.0030 (10)
O9B	0.0109 (12)	0.0119 (11)	0.0117 (13)	−0.0029 (9)	−0.0027 (10)	−0.0035 (10)
O10B	0.0117 (12)	0.0141 (12)	0.0144 (13)	0.0036 (9)	−0.0056 (10)	−0.0075 (11)
O11B	0.0114 (12)	0.0088 (11)	0.0143 (13)	−0.0008 (9)	−0.0031 (10)	−0.0023 (10)
O12B	0.0120 (12)	0.0093 (11)	0.0131 (13)	−0.0008 (9)	−0.0048 (10)	−0.0031 (10)
O13T	0.0094 (12)	0.0129 (12)	0.0203 (14)	0.0008 (9)	−0.0046 (11)	−0.0078 (11)
O14T	0.0130 (12)	0.0136 (12)	0.0145 (13)	−0.0021 (9)	−0.0032 (11)	−0.0035 (11)
O15T	0.0126 (12)	0.0166 (13)	0.0163 (14)	−0.0034 (10)	−0.0034 (11)	−0.0011 (11)
O16T	0.0163 (13)	0.0204 (13)	0.0151 (14)	0.0005 (10)	−0.0063 (11)	−0.0073 (12)
O17T	0.0141 (13)	0.0159 (13)	0.0213 (15)	0.0034 (10)	−0.0081 (11)	−0.0062 (12)
O18T	0.0148 (12)	0.0164 (12)	0.0152 (13)	−0.0002 (10)	−0.0073 (11)	−0.0070 (11)
O19T	0.0142 (13)	0.0168 (13)	0.0248 (15)	0.0045 (10)	−0.0078 (12)	−0.0104 (12)
O20T	0.0145 (13)	0.0157 (13)	0.0159 (14)	−0.0030 (10)	−0.0029 (11)	−0.0017 (11)
O21T	0.0166 (14)	0.0163 (13)	0.0166 (15)	0.0011 (10)	−0.0012 (12)	−0.0012 (12)
O22T	0.0201 (13)	0.0196 (13)	0.0138 (13)	0.0042 (10)	−0.0092 (11)	−0.0097 (11)
O23T	0.0171 (13)	0.0144 (12)	0.0156 (14)	−0.0006 (10)	−0.0069 (11)	−0.0076 (11)
O24T	0.0108 (12)	0.0106 (11)	0.0152 (13)	0.0011 (9)	−0.0050 (10)	−0.0044 (10)
O1W	0.0324 (18)	0.041 (2)	0.0307 (19)	−0.0046 (15)	0.0027 (15)	−0.0203 (17)
O2W	0.099 (4)	0.088 (3)	0.026 (2)	0.072 (3)	−0.019 (2)	−0.023 (2)
O3W	0.0254 (16)	0.0216 (15)	0.044 (2)	−0.0014 (13)	−0.0079 (15)	−0.0178 (15)
O4W	0.0189 (14)	0.0273 (16)	0.0163 (15)	−0.0046 (12)	−0.0062 (12)	0.0009 (13)
O5W	0.0227 (15)	0.0132 (13)	0.0284 (17)	0.0015 (11)	−0.0129 (13)	−0.0091 (13)
O6W	0.051 (6)	0.054 (6)	0.037 (4)	0.030 (4)	−0.032 (4)	−0.018 (4)

### Geometric parameters (Å, °)

**Table:**

Pt—O1C	1.979 (2)	Mo6—O5C	2.091 (2)
Pt—O2C	2.006 (2)	Mo6—O6C	2.286 (2)
Pt—O3C	2.034 (2)	Mo6—O11B	2.114 (2)
Pt—O4C	2.011 (2)	Mo6—O12B	1.840 (2)
Pt—O5C	1.992 (2)	Mo6—O23T	1.701 (2)
Pt—O6C	1.986 (2)	Mo6—O24T	1.756 (2)
Pt—Mo6	3.2149 (3)	K1—O10B	2.713 (3)
Mo1—Mo2	3.2140 (4)	K1—O14T^i^	2.854 (3)
Mo1—O1C	2.118 (2)	K1—O17T^ii^	2.880 (3)
Mo1—O6C	2.329 (2)	K1—O1W	3.039 (4)
Mo1—O7B	1.951 (2)	K1—O3W	2.986 (3)
Mo1—O12B	1.984 (2)	K1—O4W	2.700 (3)
Mo1—O13T	1.742 (2)	K1—O4W^iii^	2.815 (3)
Mo1—O14T	1.694 (2)	K2—O9B^iv^	2.574 (2)
Mo2—O1C	2.196 (2)	K2—O9B	2.574 (2)
Mo2—O2C	2.289 (2)	K2—O13T^v^	3.128 (3)
Mo2—O7B	1.987 (2)	K2—O13T^vi^	3.128 (3)
Mo2—O8B	1.927 (2)	K2—O18T^iv^	3.149 (3)
Mo2—O15T	1.704 (2)	K2—O18T	3.149 (3)
Mo2—O16T	1.716 (3)	K2—O24T^vi^	2.767 (2)
Mo3—O2C	2.326 (2)	K2—O24T^v^	2.767 (2)
Mo3—O3C	2.273 (3)	K3—O8B^i^	2.799 (2)
Mo3—O8B	1.936 (2)	K3—O15T^i^	2.985 (3)
Mo3—O9B	1.936 (2)	K3—O16T^vii^	2.983 (3)
Mo3—O17T	1.698 (2)	K3—O19T^viii^	3.105 (3)
Mo3—O18T	1.721 (2)	K3—O22T	3.282 (3)
Mo4—O3C	2.285 (2)	K3—O23T	2.749 (3)
Mo4—O4C	2.323 (2)	K3—O2W	2.706 (4)
Mo4—O9B	1.923 (3)	K3—O5W	3.068 (3)
Mo4—O10B	1.950 (2)	K4—O16T	2.720 (3)
Mo4—O19T	1.697 (2)	K4—O18T	2.995 (3)
Mo4—O20T	1.707 (2)	K4—O20T^iv^	2.702 (3)
Mo5—O4C	2.278 (3)	K4—O22T^ix^	2.744 (3)
Mo5—O5C	2.151 (2)	K4—O24T^v^	2.883 (3)
Mo5—O10B	1.892 (2)	K4—O1W^ix^	2.756 (4)
Mo5—O11B	2.076 (2)	K4—O6W^x^	2.866 (8)
Mo5—O21T	1.715 (2)	K4—O6W	2.687 (8)
Mo5—O22T	1.702 (2)	O6C—H6	1.28 (0)
			
O1C—Pt—O6C	83.77 (10)	O4W—K1—O3W	95.82 (9)
O1C—Pt—O5C	97.36 (10)	O10B—K1—O3W	71.65 (8)
O6C—Pt—O5C	83.63 (10)	O4W^iii^—K1—O3W	136.06 (9)
O1C—Pt—O2C	82.44 (10)	O14T^i^—K1—O3W	66.24 (8)
O6C—Pt—O2C	96.73 (10)	O17T^ii^—K1—O3W	82.07 (9)
O5C—Pt—O2C	179.57 (9)	O4W—K1—O1W	71.71 (9)
O1C—Pt—O4C	177.47 (9)	O10B—K1—O1W	72.44 (8)
O6C—Pt—O4C	98.47 (10)	O4W^iii^—K1—O1W	102.34 (9)
O5C—Pt—O4C	81.74 (10)	O14T^i^—K1—O1W	139.77 (9)
O2C—Pt—O4C	98.44 (10)	O17T^ii^—K1—O1W	145.67 (9)
O1C—Pt—O3C	95.90 (10)	O3W—K1—O1W	115.70 (9)
O6C—Pt—O3C	179.41 (9)	O9B^iv^—K2—O9B	180.000 (17)
O5C—Pt—O3C	96.90 (10)	O9B—K2—O24T^vi^	83.34 (7)
O2C—Pt—O3C	82.74 (10)	O9B—K2—O24T^v^	96.66 (7)
O4C—Pt—O3C	81.88 (10)	O9B—K2—O13T^vi^	93.58 (7)
O14T—Mo1—O13T	105.35 (12)	O9B—K2—O18T^iv^	123.48 (7)
O14T—Mo1—O7B	100.55 (11)	O9B^iv^—K2—O18T	123.48 (7)
O13T—Mo1—O7B	96.64 (11)	O9B—K2—O18T	56.52 (7)
O14T—Mo1—O12B	96.89 (11)	O24T^vi^—K2—O18T	107.85 (7)
O13T—Mo1—O12B	96.56 (11)	O24T^v^—K2—O18T	72.15 (7)
O7B—Mo1—O12B	154.46 (10)	O13T^v^—K2—O18T	119.67 (6)
O14T—Mo1—O1C	96.38 (10)	O13T^vi^—K2—O18T	60.33 (6)
O13T—Mo1—O1C	158.00 (10)	O18T^iv^—K2—O18T	180.00 (14)
O7B—Mo1—O1C	75.67 (9)	O2W—K3—O23T	87.40 (12)
O12B—Mo1—O1C	84.01 (10)	O2W—K3—O8B^i^	101.70 (15)
O14T—Mo1—O6C	165.54 (10)	O23T—K3—O8B^i^	135.26 (8)
O13T—Mo1—O6C	86.23 (10)	O2W—K3—O16T^vii^	68.29 (11)
O7B—Mo1—O6C	86.39 (10)	O23T—K3—O16T^vii^	112.47 (8)
O12B—Mo1—O6C	72.78 (9)	O2W—K3—O15T^i^	117.98 (11)
O1C—Mo1—O6C	72.89 (9)	O23T—K3—O15T^i^	151.29 (8)
O15T—Mo2—O16T	106.40 (12)	O2W—K3—O5W	121.75 (11)
O15T—Mo2—O8B	98.22 (11)	O23T—K3—O5W	64.76 (7)
O16T—Mo2—O8B	100.68 (11)	O8B^i^—K3—O5W	73.57 (7)
O15T—Mo2—O7B	101.30 (11)	O16T^vii^—K3—O5W	168.39 (8)
O16T—Mo2—O7B	92.82 (11)	O15T^i^—K3—O5W	107.09 (7)
O8B—Mo2—O7B	152.03 (10)	O2W—K3—O19T^viii^	66.76 (11)
O15T—Mo2—O1C	92.44 (11)	O23T—K3—O19T^viii^	63.34 (7)
O16T—Mo2—O1C	158.60 (10)	O5W—K3—O19T^viii^	55.12 (7)
O8B—Mo2—O1C	86.22 (9)	O2W—K3—O22T	110.14 (14)
O7B—Mo2—O1C	73.19 (9)	O23T—K3—O22T	59.79 (7)
O15T—Mo2—O2C	161.42 (11)	O8B^i^—K3—O22T	145.97 (7)
O16T—Mo2—O2C	91.12 (11)	O16T^vii^—K3—O22T	71.25 (7)
O8B—Mo2—O2C	71.88 (9)	O15T^i^—K3—O22T	96.63 (7)
O7B—Mo2—O2C	83.57 (9)	O5W—K3—O22T	98.56 (7)
O1C—Mo2—O2C	71.63 (9)	O19T^viii^—K3—O22T	123.13 (7)
O17T—Mo3—O18T	106.56 (12)	O6W—K4—O20T^iv^	86.85 (17)
O17T—Mo3—O8B	98.83 (11)	O6W—K4—O16T	100.30 (17)
O18T—Mo3—O8B	101.53 (11)	O20T^iv^—K4—O16T	163.39 (9)
O17T—Mo3—O9B	101.37 (11)	O6W—K4—O22T^ix^	72.57 (17)
O18T—Mo3—O9B	97.67 (11)	O20T^iv^—K4—O22T^ix^	112.52 (8)
O8B—Mo3—O9B	146.75 (10)	O16T—K4—O22T^ix^	84.01 (8)
O17T—Mo3—O3C	99.47 (11)	O6W—K4—O1W^ix^	125.74 (17)
O18T—Mo3—O3C	153.10 (10)	O20T^iv^—K4—O1W^ix^	75.80 (9)
O8B—Mo3—O3C	80.67 (10)	O16T—K4—O1W^ix^	110.80 (9)
O9B—Mo3—O3C	70.18 (9)	O22T^ix^—K4—O1W^ix^	68.01 (9)
O17T—Mo3—O2C	166.65 (11)	O6W—K4—O6W^x^	22.7 (3)
O18T—Mo3—O2C	84.31 (10)	O20T^iv^—K4—O6W^x^	109.37 (17)
O8B—Mo3—O2C	70.90 (9)	O16T—K4—O6W^x^	77.87 (16)
O9B—Mo3—O2C	84.41 (10)	O22T^ix^—K4—O6W^x^	66.98 (16)
O3C—Mo3—O2C	70.96 (9)	O1W^ix^—K4—O6W^x^	132.79 (17)
O19T—Mo4—O20T	106.07 (13)	O6W—K4—O24T^v^	97.28 (17)
O19T—Mo4—O9B	101.28 (12)	O20T^iv^—K4—O24T^v^	80.55 (8)
O20T—Mo4—O9B	98.83 (12)	O16T—K4—O24T^v^	83.66 (8)
O19T—Mo4—O10B	98.77 (11)	O22T^ix^—K4—O24T^v^	162.32 (8)
O20T—Mo4—O10B	100.44 (12)	O1W^ix^—K4—O24T^v^	128.57 (9)
O9B—Mo4—O10B	147.02 (10)	O6W^x^—K4—O24T^v^	98.00 (16)
O19T—Mo4—O3C	95.89 (11)	O6W—K4—O18T	164.77 (16)
O20T—Mo4—O3C	157.14 (10)	O20T^iv^—K4—O18T	102.64 (8)
O9B—Mo4—O3C	70.14 (9)	O16T—K4—O18T	67.51 (7)
O10B—Mo4—O3C	82.01 (10)	O22T^ix^—K4—O18T	113.50 (8)
O19T—Mo4—O4C	163.39 (11)	O1W^ix^—K4—O18T	68.76 (9)
O20T—Mo4—O4C	88.94 (11)	O6W^x^—K4—O18T	144.85 (16)
O9B—Mo4—O4C	82.95 (10)	O24T^v^—K4—O18T	72.99 (7)
O10B—Mo4—O4C	70.94 (9)	Pt—O1C—Mo1	105.46 (10)
O3C—Mo4—O4C	70.23 (9)	Pt—O1C—Mo2	104.98 (10)
O22T—Mo5—O21T	106.70 (13)	Mo1—O1C—Mo2	96.30 (9)
O22T—Mo5—O10B	101.84 (11)	Pt—O2C—Mo2	100.81 (10)
O21T—Mo5—O10B	102.99 (11)	Pt—O2C—Mo3	102.68 (10)
O22T—Mo5—O11B	97.58 (11)	Mo2—O2C—Mo3	92.77 (9)
O21T—Mo5—O11B	89.09 (11)	Pt—O3C—Mo3	103.60 (10)
O10B—Mo5—O11B	152.95 (10)	Pt—O3C—Mo4	104.25 (10)
O22T—Mo5—O5C	91.56 (11)	Mo3—O3C—Mo4	94.06 (9)
O21T—Mo5—O5C	154.90 (10)	Pt—O4C—Mo5	100.24 (10)
O10B—Mo5—O5C	89.52 (10)	Pt—O4C—Mo4	103.64 (10)
O11B—Mo5—O5C	71.17 (9)	Mo5—O4C—Mo4	92.53 (9)
O22T—Mo5—O4C	162.98 (11)	Pt—O5C—Mo6	103.86 (10)
O21T—Mo5—O4C	90.31 (11)	Pt—O5C—Mo5	105.34 (10)
O10B—Mo5—O4C	72.93 (9)	Mo6—O5C—Mo5	105.12 (10)
O11B—Mo5—O4C	83.03 (9)	Pt—O6C—Mo6	97.38 (10)
O5C—Mo5—O4C	72.45 (9)	Pt—O6C—Mo1	97.89 (10)
O23T—Mo6—O24T	104.61 (11)	Mo6—O6C—Mo1	90.69 (8)
O23T—Mo6—O12B	103.60 (11)	Mo1—O7B—Mo2	109.41 (11)
O24T—Mo6—O12B	103.93 (11)	Mo2—O8B—Mo3	119.78 (12)
O23T—Mo6—O5C	92.37 (11)	Mo2—O8B—K3^i^	101.44 (9)
O24T—Mo6—O5C	154.05 (10)	Mo3—O8B—K3^i^	137.03 (11)
O12B—Mo6—O5C	90.63 (10)	Mo4—O9B—Mo3	119.62 (12)
O23T—Mo6—O11B	93.94 (11)	Mo5—O10B—Mo4	119.84 (12)
O24T—Mo6—O11B	87.50 (10)	Mo5—O11B—Mo6	107.00 (11)
O12B—Mo6—O11B	155.64 (10)	Mo6—O12B—Mo1	118.20 (12)
O5C—Mo6—O11B	71.63 (9)	H1A—O1W—H1B	103 (4)
O23T—Mo6—O6C	166.81 (10)	H2A—O2W—H2B	105 (4)
O24T—Mo6—O6C	88.05 (10)	H3A—O3W—H3B	107 (3)
O12B—Mo6—O6C	76.34 (10)	H4A—O4W—H4B	109 (4)
O5C—Mo6—O6C	74.46 (9)	H5A—O5W—H5B	105 (3)
O11B—Mo6—O6C	82.73 (9)	H6A—O6W—H6B	101 (10)
O4W—K1—O10B	131.27 (9)		

Symmetry codes: (i) −*x*+1, −*y*+1, −*z*+1; (ii) −*x*, −*y*+1, −*z*+1; (iii) −*x*, −*y*+1, −*z*+2; (iv) −*x*, −*y*, −*z*+1; (v) −*x*+1, −*y*, −*z*+1; (vi) *x*−1, *y*, *z*; (vii) *x*, *y*, *z*+1; (viii) *x*+1, *y*, *z*; (ix) *x*, *y*, *z*−1; (x) −*x*+1, −*y*, −*z*.

### Hydrogen-bond geometry (Å, °)

**Table:**

*D*—H···*A*	*D*—H	H···*A*	*D*···*A*	*D*—H···*A*
O2C—H2···O24T^v^	0.87 (3)	1.70 (3)	2.561 (3)	168 (5)
O3C—H3···O5W^i^	0.89 (3)	1.67 (3)	2.552 (4)	177 (5)
O4C—H4···O13T^v^	0.89 (5)	1.67 (5)	2.562 (3)	176 (5)
O6C—H6···O6C^v^	1.28	1.28	2.553 (3)	180
O11B—H11···O7B^v^	0.90 (5)	1.93 (5)	2.826 (3)	172 (4)
O1W—H1A···O13T^xi^	0.84 (3)	2.27 (4)	3.014 (4)	149 (5)
O1W—H1B···O21T^xii^	0.89 (3)	2.22 (4)	3.067 (4)	159 (6)
O2W—H2A···O7B^vii^	0.86 (3)	2.10 (4)	2.906 (5)	157 (7)
O2W—H2B···O21T^xiii^	0.83 (3)	2.49 (5)	3.038 (5)	125 (5)
O3W—H3A···O1C^i^	0.86 (3)	2.01 (3)	2.811 (4)	154 (5)
O3W—H3B···O5C	0.83 (3)	2.24 (3)	2.973 (4)	147 (4)
O4W—H4A···O15T^i^	0.81 (3)	2.03 (3)	2.839 (4)	175 (5)
O4W—H4B···O18T^vii^	0.84 (3)	2.11 (3)	2.938 (4)	168 (4)
O5W—H5A···O19T^viii^	0.86 (3)	2.14 (3)	2.856 (4)	141 (4)
O5W—H5B···O3W^i^	0.83 (2)	1.99 (3)	2.787 (4)	160 (4)
O6W—H6A···O22T^v^	0.85 (3)	2.41 (8)	3.097 (7)	138 (11)

Symmetry codes: (v) −*x*+1, −*y*, −*z*+1; (i) −*x*+1, −*y*+1, −*z*+1; (xi) *x*−1, *y*, *z*+1; (xii) −*x*, −*y*, −*z*+2; (vii) *x*, *y*, *z*+1; (xiii) −*x*+1, −*y*, −*z*+2; (viii) *x*+1, *y*, *z*.
